# Extending bioacoustic monitoring of birds aloft through flight call localization with a three‐dimensional microphone array

**DOI:** 10.1002/ece3.2447

**Published:** 2016-09-12

**Authors:** Phillip M. Stepanian, Kyle G. Horton, David C. Hille, Charlotte E. Wainwright, Phillip B. Chilson, Jeffrey F. Kelly

**Affiliations:** ^1^ School of Meteorology University of Oklahoma Norman OK USA; ^2^ Advanced Radar Research Center University of Oklahoma Norman OK USA; ^3^ Department of Biology Oklahoma Biological Survey University of Oklahoma Norman OK USA; ^4^ Present address: Department of Agroecology Rothamsted Research Harpenden Hertfordshire AL5 2JQ UK

**Keywords:** acoustics, aeroecology, recording, triangulation

## Abstract

Bioacoustic localization of bird vocalizations provides unattended observations of the location of calling individuals in many field applications. While this technique has been successful in monitoring terrestrial distributions of calling birds, no published study has applied these methods to migrating birds in flight. The value of nocturnal flight call recordings can increase with the addition of three‐dimensional position retrievals, which can be achieved with adjustments to existing localization techniques. Using the time difference of arrival method, we have developed a proof‐of‐concept acoustic microphone array that allows the three‐dimensional positioning of calls within the airspace. Our array consists of six microphones, mounted in pairs at the top and bottom of three 10‐m poles, arranged in an equilateral triangle with sides of 20 m. The microphone array was designed using readily available components and costs less than $2,000 USD to build and deploy. We validate this technique using a kite‐lofted GPS and speaker package, and obtain 60.1% of vertical retrievals within the accuracy of the GPS measurements (±5 m) and 80.4% of vertical retrievals within ±10 m. The mean Euclidian distance between the acoustic retrievals of flight calls and the GPS truth was 9.6 m. Identification and localization of nocturnal flight calls have the potential to provide species‐specific spatial characterizations of bird migration within the airspace. Even with the inexpensive equipment used in this trial, low‐altitude applications such as surveillance around wind farms or oil platforms can benefit from the three‐dimensional retrievals provided by this technique.

## 1 Introduction

A core problem for research on nocturnal migration for the past century has been validation of abundance, distribution, and species composition of animals aloft (Kunz et al., 2008). Recent developments in remote sensing methods can provide a subset of information on bulk abundance, distribution, phenology, identity, and behavior of migrants, but a complementary suite of sensors is needed to obtain the full set of these data (Horton, Shriver, & Buler, 2015). Techniques for migration monitoring have incorporated observations from radar, thermal imaging, and audio recordings, but only the analysis of night flight calls can provide taxonomic identity of migrants. For this reason, nocturnal flight call data are often used to provide species composition or relative abundance estimates in concert with more robust methods of estimating total distribution and abundance of migrants (Farnsworth, Gauthreaux, & Van Blaricom, 2004; Hüppop, Dierschke, Exo, Fredrich, & Hill, 2006). We present a method for estimating the position of birds producing nocturnal flight calls, which will increase their value for describing the spatiotemporal distribution of these species‐specific vocalizations.

Acoustic observations of nocturnal flight calls have long been a source of information on the presence and identity of birds in the airspace (e.g., Farnsworth, 2005; Libby, 1899). The development of amplification and recording devices propelled acoustic methods into regular use in avian field studies (e.g., Graber & Cochran, 1959). Some applications include the use of acoustic proxies for abundance (e.g., Farnsworth et al., 2004), as well as regionally distributed recording stations for broad‐scale distribution studies (e.g., Evans & Mellinger, 1999). Recent advances in wireless electronics and digital recording have resulted in sophisticated audio processing techniques (Blumstein et al., 2011), including automated call detection (Potamitis, Ntalampiras, Jahn, & Riede, 2014), recognition (e.g., Baker & Logue, 2003; Cortopassi & Bradbury, 2000; Kogan & Margoliash, 1998), and localization.

Acoustic localization (sometimes referred to as “triangulation”) is the process of identifying the source location of sounds using recordings from multiple time‐synchronized microphones (Blumstein et al., 2011). Bioacoustic localization of calling animals has been developed theoretically (e.g., Magyar, Schleidt, & Miller, 1978; Spiesberger, 2001, 2005; Spiesberger & Fristrup, 1990) and demonstrated in laboratory and field trials (e.g., Gaudette & Simmons, 2014). While the utility of these techniques for wildlife monitoring has been illustrated, it is often the case that applications are limited in spatial extent or dimension. For example, acoustic localization of bats is typically conducted indoors within the quiet confines of a laboratory setting (e.g., Barchi, Knowles, & Simmons, 2013; Falk, Jakobsen, Surlykke, & Moss, 2014). In outdoor field applications, the acoustic recorders must be in close proximity to the flying bats to ensure detectability of their ultrasonic calls, resulting in relatively small spatial coverage (e.g., Fujioka, Aihara, Sumiya, Aihara, & Hiryu, 2016). The attenuation of such high‐frequency calls can be quite severe, with studies showing maximum call detection ranges on the order of several meters in some cases (Jenson & Miller, 1999; Stilz & Schnitzler, 2012).

While the relatively lower audio frequencies of bird calls are less affected by these range‐limiting effects, all previous studies have been limited exclusively to terrestrial or near‐terrestrial environments. Applications that have used call localization to retrieve the ground positions of birds include those by Magyar et al. (1978) on Bobwhite Quails (*Colinus virginianus*), Collier, Kirschel, and Taylor (2010) on the Mexican Antthrush (*Formicarius moniliger*), and, most recently, the multiyear study by Frommolt and Tauchert (2014) on the Eurasian Bittern (*Botaurus stellaris*). Similar to these studies, both Wang et al. (2005) and Wilson, Battiston, Brzustowski, and Mennill (2013) describe all calls and retrievals as occurring in the same horizontal plane, indicating two‐dimensional localization. Several studies have retrieved vocalizations that are representative of birds perched above ground level (e.g., McGregor et al., 1997; Mennill, Battiston, Wilson, Foote, & Doucet, 2012; Mennill, Burt, Fristrup, & Vehrencamp, 2006; Spiesberger, 1999); however, the maximum retrieval heights in these studies did not exceed 3 m. From our investigations, no published study has localized bird calls above 13.5 m (Ali et al., 2009) or in migratory flight within the airspace.

The extension of bioacoustic localization of birdcalls to three‐dimensional space can provide explicit surveillance of calling nocturnal migrants. To demonstrate the utility of these techniques, we constructed an acoustic microphone array as a flight call localization proof of concept. In the following sections, we describe the construction of the array, audio processing techniques for localization, and retrieval validation. We also describe the challenges associated with deploying a setup of this type in the field and offer practical considerations that should be taken into account in designing such experiments.

## 2 Materials and Methods

### 2.1 Computational methods

Several techniques exist for extracting sound source locations from multiple recordings (Blumstein et al., 2011). Many of these techniques have been developed for diverse applications ranging from acoustic aircraft surveillance (Blumrich & Altmann, 2000) to enemy gunshot positioning (Ferguson, Criswick, & Lo, 2002). For this study, we focus on the time difference of arrival (TDOA) method, which has been successfully transitioned to a number of biological applications including monitoring marine (Clark & Ellison, 2000; Giraudet & Glotin, 2006; Muanke & Niezrecki, 2007; Nosal, 2013) and terrestrial wildlife (Collier et al., 2010; Magyar et al., 1978; Spiesberger & Fristrup, 1990). The fundamental TDOA technique was developed for radio navigation in the early 1970s (Schmidt, 1972; Van Etten, 1970) and has been subsequently applied to several bioacoustic software packages [e.g., Raven Pro (Cornell Lab of Ornithology, Ithaca, NY, USA); Avisoft‐SASLab Pro (Avisoft Bioacoustics, Berlin, Germany); SIGNAL (Engineering Design, Belmont, MA, USA); ArrayGUI (J. Burt, Seattle, WA, USA); Sound Finder (Wilson et al., 2013)]. The basic TDOA workflow that we apply is as follows:

1. We record six synchronized channels of audio from the microphone array (detailed in the following section).
2. We manually screen the recordings to ensure that each call is detected on all of the six channels.
3. We use a MATLAB (2010) software package (The MathWorks Inc., Natick, MA) that was written ad hoc to calculate the temporal cross‐correlation of the filtered audio waveforms from each channel to obtain the arrival time lags (following Spiesberger & Fristrup, 1990).
4. We calculate the sound source location from the six time lags using the set of equations presented by Spiesberger (2001), implemented in MATLAB, The MathWorks Inc.

The results that are presented through the duration of this paper were obtained using this workflow.

### 2.2 Acoustic array design

**Figure 1:**
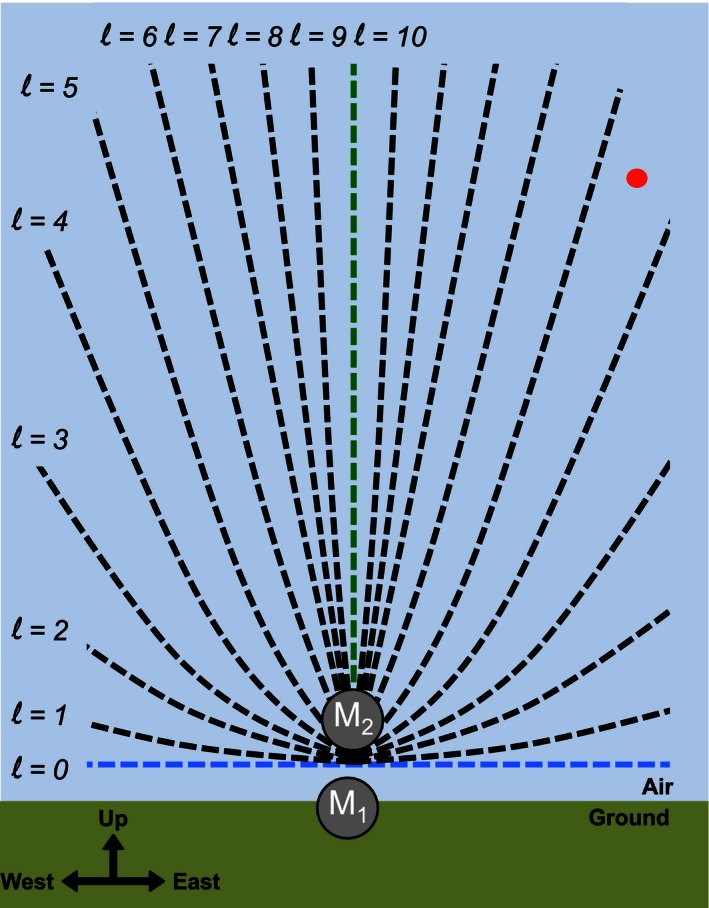
Schematic of two vertically separated microphones and all possible hyperboloids for *l*_max_ = 10. The red circle indicates the sound source location of a calling bird

The basic hardware requirement for 3D TDOA localization is a distributed network of five or more time‐synchronized recording devices (Spiesberger, 2001); however, it is the placement of these microphones combined with the recorder sample rate that determines whether practical 3D localization can be achieved. To demonstrate this dependence, consider a vertical tower with a microphone (M_1_) located at the base and a second microphone (M_2_) located 154 cm directly above M_1_ (Fig. 1). We will call this separation distance between the microphones *d*. Both microphones are synchronized and recording at a rate of 22,050 samples per second (i.e., *τ* = 22,050 Hz), and the atmospheric speed of sound, *v*, is 340 m/s. In this case, the minimum distinguishable distance between consecutive recorded samples is Δ*d* = *v*/*τ* = 15.4 cm. When a flying bird calls (Fig. 1, red circle), the sound will eventually arrive at both microphones, and the offset number of recording samples, or lag, between the arrivals can be computed. The maximum possible lag, *l*_max_, will occur when the call is directly above the tower (Fig. 1, green line) and is equal to the maximum lag samples that fit between M_1_ and M_2_. That is, *l*_max_ = *d/*Δ*d* = 10 samples. Of course, the minimum possible lag is zero, which will occur when the call is located on the plane equidistant from the two microphones (Fig. 1, blue line). As a result, there are only 11 possible lags that can occur l = 0, 1, 2, 3, 4, 5, 6, 7, 8, 9, 10. Each of lags 1 through 9 creates a unique hyperboloid passing between the vertical line and the horizontal plane (Fig. 1, black curves). The more black curves that exist, the greater the number of possible localization outcomes, and therefore, the higher the possible retrieval accuracy. The number of black curves will always equal (*l*_max_−1), so there are only two ways to increase accuracy: increase the audio sampling rate or increase the distance between microphones. In this example, no hyperboloid passes through the location of the bird, and so the retrieval must select one of the neighboring hyperboloids. This necessary deviation from the true bird location results in retrieval error. By doubling the distance between microphones (*d *=* *308 cm), *l*_max_ will increase to 20 samples, and the number of hyperboloid solutions will double, effectively placing an additional black curve between each existing one and decreasing the error in the bird location solution.

**Figure 2:**
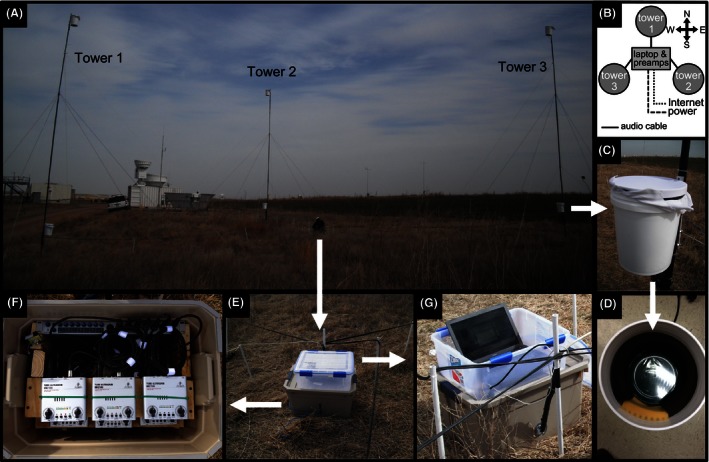
Acoustic array setup and components. (A) Photograph of array deployment in Billings, Oklahoma. (B) Schematic of array layout. (C) Close‐up on one microphone enclosure with protective cloth cover. (D) Inside of microphone enclosure revealing foam baffling surrounding flowerpot microphone. (E) Central enclosures holding amplifiers (bottom) and laptop (top). (F) Inside of amplification enclosure. (G) Laptop for data acquisition and storage

In short, accurate three‐dimensional localization requires sufficient microphone height diversity in the array layout (An & Chen, 2015). Many microphone arrays are distributed with all microphones at the same or similar heights, resulting in high retrieval uncertainty in altitude (e.g., Wang et al., 2005; Wilson et al., 2013). In fact, the best demonstrations of 3D localization have been performed in aquatic environments using hydrophones suspended at different depths below the ocean surface (e.g., An & Chen, 2015; Wahlberg, Møhl, & Madsen, 2001). With this inherent limitation in mind, we increased the maximum potential accuracy for altitudinal retrievals by increasing vertical microphone separations using three 9.14‐m towers. Each tower was constructed from three connected 10‐foot segments of schedule 40 black iron pipe using the standard pipe couplings and was held upright by several guy wires and rebar stakes (Fig. 2A). The bottom two segments of pipe were each one inch in diameter, while the top segment was reduced to 0.75 inch. Microphones were secured to the top and bottom of each tower with metal L‐brackets, with towers arranged in an equilateral triangle with vertices 20 m apart (Fig. 2B). Tower spacing was achieved using several tape measures simultaneously pulled taut between vertices. Rather than placing the lower microphones directly on the ground, they were secured at approximately 1.5 m high on the tower to avoid infestations by rodents and insects, as well as to mitigate noise from insects on the ground. In this configuration, we were able to install the array with only two people.

Microphones were designed following Evans and Mellinger (1999), using a Knowles Electret EK3132 condenser microphone element mounted on a 16.5‐cm plate and housed within a 5‐gallon plastic pale (Fig. 2C). To reduce the ground‐level noise contamination, the microphone housing was lined with noise canceling acoustic foam (Fig. 2D). Audio cables connected the six microphones to the central recording hardware, housed in weatherproof containers (Fig. 2E). Each individual microphone was amplified using a Behringer Tube Ultragrain MIC100 preamplifier and routed to a PreSonus DigiMax D8 preamplifier (Fig. 2F). The resulting amplified ADAT format audio signals were fed into a laptop via a FireWire connection, digitized using Raven Pro v1.4 running on Windows 7 operating system, saved in six‐channel .wav files every 5 min, and sent to a remote computer for storage over an Internet connection (Fig. 2G).

This array design was based on material availability, cost, simplicity of construction, and ease of field deployment rather than optimized theory and should be viewed as a lower limit on potential capabilities. Our final array (microphones, cables, amplifiers, towers, supports, and mounts) cost less than $2,000 (USD) to construct and deploy, and used readily available hardware.

## 3 Validation Using Kite‐Lofted Speakers and GPS

**Figure 3:**
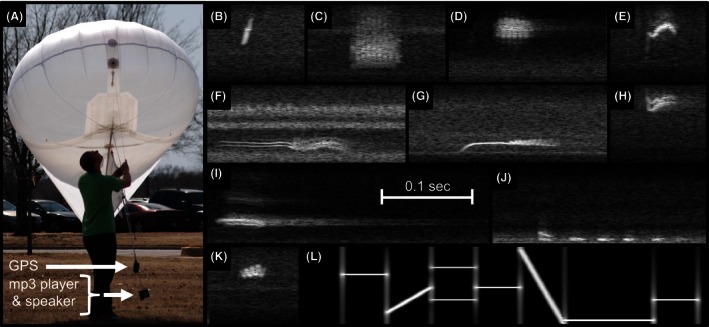
Validation using helikite and test samples. (A) Helikite with attached GPS recorder, mp3 player, and speakers. (B–L) Spectrograms for test sample recordings from Evans and O'Brien (2002): (B) Black‐throated Blue Warbler, (C) Dickcissel, (D) Indigo Bunting, (E) Ovenbird, (F) Summer Tanager, (G) Swainson's Thrush, (H) Vesper Sparrow, (I) Wood Thrush, (J) Yellow‐billed Cuckoo, (K) Yellow Warbler, and (L) synthetic signal. Inset time scale in (I) is valid for all call samples, and all frequency axes range from 0 to 10 kHz

An initial validation experiment was conducted at the Oklahoma Biological Survey, located at the University of Oklahoma in Norman, Oklahoma, USA. The experiment site is a grass field in a suburban area and is close to several roads and buildings. As a first proof of concept, the microphone array was tested on generated calls at known locations aloft. This was achieved by attaching a small speaker (AUVIO model #4000038; 1.5 W), mp3 player (Philips GoGear SA2315), and GPS unit (Garmin GPSmap 62st) to a helium balloon‐kite hybrid (hereafter helikite; Fig. 3A). The mp3 player was used to broadcast a series of eleven prerecorded samples at 3‐s intervals, including flight calls from ten bird species (from Evans & O'Brien, 2002), and one synthetic tone sequence. The ten flight calls were chosen to cover a wide range of frequencies, durations, and bandwidths, and are illustrated in the spectrograms shown in Fig. 3B–K. Calls were broadcast in their original, unaltered .wav format. The synthetic tone is depicted in Fig. 3L. This audio loop was played continuously throughout the experiment, while the speaker was moved throughout the airspace by raising, lowering, and walking with the helikite tether line. The use of a helikite, as opposed to a standard balloon, provides enhanced stability in light winds, but does not itself rely on wind to remain aloft. The speaker package was suspended by a line approximately 1 m below the helikite in a general downward direction (Fig. 3A). Unfortunately, this configuration also enabled the speaker to swing with the movements of the helikite, sometimes directing broadcasts away from the microphone array. The collocated GPS made measurements of the speaker location approximately every 7 s. The maximum horizontal and vertical distances from the center of the microphone array to the helikite were 105 and 140 m, respectively. The maximum Euclidean distance from the center of the array to the helikite was 175 m. Upon completion of the field experiment, the localization algorithm was run on all recorded calls and compared to the GPS measurements. As calls were broadcast at a fixed interval and set pattern, the time of each call is known a priori, and bandpass filters for each call were used to improve detectability.

**Figure 4:**
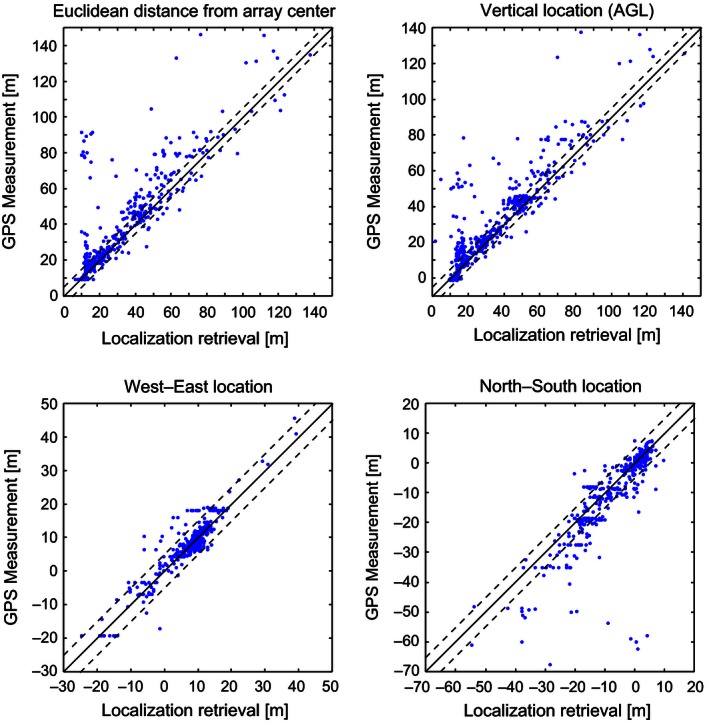
Comparison of localization results to GPS measurements. (upper left) Comparison of total Euclidean distance from array center. (upper right) Altitudinal retrieval comparisons in height above ground level. (bottom left) Longitudinal retrieval comparisons. (bottom right) Latitudinal retrieval comparisons. Detected call sample size was *n* = 474. The solid line denotes the one‐to‐one boundary. The region bounded by the dashed lines indicates the reported measurement uncertainty of the GPS unit (±5 m for *x*,* y*,* z*; ±8.66 m for Euclidian distance)

The resulting localization retrievals were compared to the GPS “truth” measurements to determine the localization errors by subtracting the GPS components from the retrieval components (Fig. 4). Because the GPS unit reports with 5 m accuracy, localization results within 5 m of the GPS are considered perfect retrievals. Comparison with the GPS reveals high localization retrieval accuracy for the detected calls, with 60.1% of vertical retrievals having accuracy within the uncertainty of the GPS unit and 80.4% of vertical retrievals within ±10 m. The errors associated with vertical retrievals are typically underestimates (Fig. 4, right; occurring in the upper left half of the one‐to‐one line), suggesting an influence of ground‐based noise sources creating a downward bias. More specifically, these cases of near‐ground retrievals can be attributed to insects (i.e., crickets, grasshoppers) that produce sounds more intense than the broadcast birdcalls. Due to the small, lightweight speaker, we could only reliably detect calls up to approximately 90 m above ground level before the signal extinguished into the ambient noise. In several cases, calls were still audible above 90 m, likely due to the favorable direction the speaker was pointing as it broadcast the call. In these cases, the calls were still detected in all microphones and could be localized, with a maximum retrieval height of approximately 130 m above ground level (Fig. 4).

**Figure 5:**
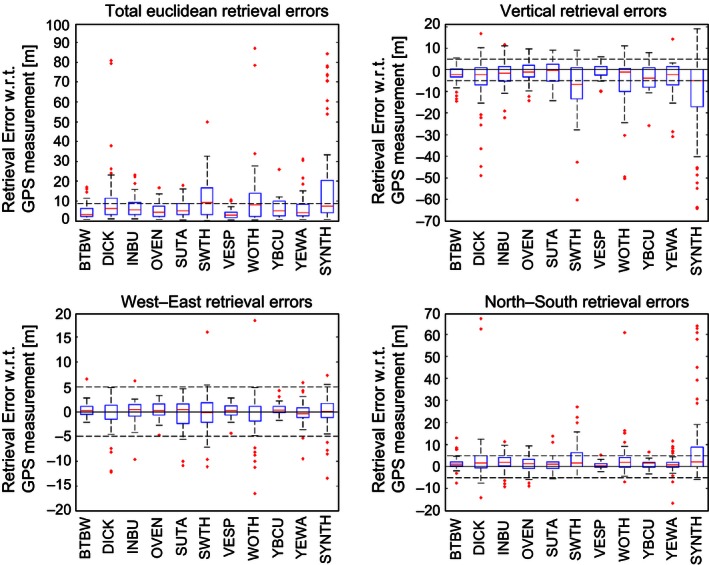
Call‐specific errors. (upper left) Total retrieval errors. (upper right) Altitudinal retrieval errors. (bottom left) Longitudinal retrieval errors. (bottom right) Latitudinal retrieval errors. Red dots indicate outliers. The region bounded by the dashed lines indicates the reported measurement uncertainty of the GPS unit (±5 m for *x*,* y*,* z*; ±8.66 m for Euclidian distance). Alpha codes correspond to calls listed in Figure 3. Detectable call sample sizes are *n* = 37, 55, 41, 44, 45, 50, 33, 49, 15, 41, 64

Additionally, these retrieval errors are summarized in terms of call‐specific variations (Fig. 5). Considering the distribution of these errors, it is clear that there were consistent differences in the retrieval performance for the various calls. A dominant factor in these retrievals is the acoustic frequency of the underlying call. The atmosphere acts as a low‐pass acoustic filter, and so low‐frequency calls should attenuate the least along their path (Horton, Stepanian, Wainwright, & Tegeler, 2015). As a result, we would expect that low‐frequency calls should be the most detectable, and in an atmosphere free of background noise, this would be true. However, ambient noise is also preferentially attenuated at higher frequencies, resulting in greater noise amplitudes at lower‐frequency bands. As such, some low‐frequency flight calls such as the Yellow‐billed Cuckoo reside in this elevated noise region (see Fig. 3J) and can be effectively indistinguishable from background noise. The practical effect of this enhanced noise is a general lack of calls that have sufficient signal‐to‐noise ratios to be detectable, resulting in the smallest sample size (Fig. 5, YBCU; *n* = 15). Conversely, the impulse‐like call of the Black‐throated Blue Warbler (Fig. 3B) is high enough in frequency to avoid the elevated low‐frequency noise levels, yielding exceptionally good retrievals (Fig. 5, BTBW). Similar arguments apply for the Ovenbird (Figs 3E and 5, OVEN), Indigo Bunting (Figs 3D and 5, INBU), Vesper Sparrow (Figs 3H and 5, VESP), and Yellow Warbler (Figs 3K and 5, YEWA), all of which have calls in a similar frequency band. Most surprisingly, it was the synthetic tone sequence (Figs 3L and 5, SYNTH) that yielded the worst retrievals. It is likely that the tone sequence was more conspicuous during manual spectrogram screening, which leads to its detection in lower signal‐to‐noise ratios. This explanation is supported by the higher detected sample size (*n* = 64) and would result in an increased number of retrievals that yield ground‐based noise sources.

## 4 Discussion

A recent horizon scan of current global conservation issues has highlighted the potential capabilities of passive acoustic surveillance for monitoring wildlife in terrestrial and aquatic environments (Sutherland et al., 2016), and advancements in data analysis will enable future acoustic networks to characterize the environmental soundscape near continuously (Servick, 2014). As the effects of human development continue to push farther into the airspace, there is an increasing demand to identify interactions and potential wildlife conflicts aloft. We suggest that passive acoustic localization is one such method for characterizing the airspace usage by calling animals in flight.

Acoustic flight call recordings can be compared to other remote sensing measurements such as radar or thermal images to better characterize animals in the airspace (Farnsworth et al., 2004; Horton, Shriver et al. 2015; Hüppop et al., 2006; Larkin, Evans, & Diehl, 2002). It is generally the case, however, that a recorded call cannot be directly attributed to a specific animal in other observations. For example, a flight call may be recorded while several birds are observed flying overhead, but it is usually unclear which bird uttered the call. Localization of the calls can solve this problem by providing the source position of the sound.

The localization results of our validation experiment are encouraging, with accurate retrievals as high as 130 m above ground level (Fig. 4). Similar studies have noted that artificially broadcasted calls can be much lower in amplitude than those emitted from actual birds (Ali et al., 2009), and our need to keep the speaker light exacerbated this effect. As a result, our inability to detect calls above 90 m was the dominant limitation to the validation experiment, rather than the localization technique itself. We believe that the errors recorded within our height range are still characteristic of potential retrievals higher aloft, provided similar atmospheric conditions. Admittedly, the only way to prove this accuracy at higher altitudes would require a more powerful speaker that can replicate true call amplitudes and a much larger balloon. Nonetheless, the ability to monitor the lowest 100 m of the airspace is still a promising potential, especially considering the low cost of the materials employed.

In general, the error in the retrieved vertical position is greater than the error in the retrieved horizontal location (Fig. 5). This is due to the issue illustrated in Fig. 1, as the horizontal spacing of the microphone array is much wider (~20 m) than the vertical spacing (~7.5 m). That is, there are more distinct hyperboloids of possible call locations in the horizontal plane than in the vertical plane. Further separation of the microphones in the vertical would have likely yielded improved retrievals in call altitude, but this was limited by the size of the towers.

In future trials, more effort should be devoted to baffling insect and wind noises. It may also be necessary to choose study sites with natural or purpose‐built windbreaks to mitigate these noise sources. Similarly, care must be taken to secure any loose wires running up the towers such that they do not blow in the wind and cause additional noise. Several future additions to the call retrieval technique have the potential to enhance the overall method. At higher altitudes, the propagation of bird calls will have a greater atmospheric dependence (Horton, Stepanian et al., 2015). Factors influencing propagation of calls include the variable speed of sound in regions of vertical temperature gradients and call drift from winds. Generally, these local meteorological measurements will not be available, motivating retrieval techniques that can account for these effects. Work by Spiesberger (1999, 2005) demonstrates two methods that can solve for atmospheric conditions as well as call sources. Another addition would be the use of acoustic self‐surveys as in Collier et al. (2010). By periodically transmitting an acoustic impulse from a known location, the exact microphone positions can be regularly surveyed to yield better retrievals. This process would be especially beneficial in long‐term field deployments when microphone locations may slowly change in time. For example, as guy wires gradually stretch and are retightened, towers can lean slightly off vertical, resulting in horizontal changes in microphone locations – especially at the top of the tower. Regular acoustic self‐surveys can mitigate this effect. Finally, while we describe our custom array design, the basic tower concept can be applied to Wildlife Acoustics Songmeters (Mennill et al., 2012) and Sound Finder (Wilson et al., 2013) for off‐the‐shelf operation.

The ability to connect flight calls with their location in the airspace adds value to bioacoustic recordings. These data can provide species‐specific altitudinal distributions of migrants, and their transitions within and across nights and seasons. Measurements of altitudinal preferences during migration can be compared to meteorological conditions to deduce the decision‐making processes of animals on the move. Furthermore, the ability to provide an exact position of calling birds can allow better risk assessments; for example, one could determine whether calling birds are flying above, below, or within the height of wind turbine rotors or other aerial hazards. Overall, the potential capability of flight call localization in migration monitoring motivates further studies into the development and refinement of such techniques.

## Funding Information

Division of Emerging Frontiers (Grant/Award Number: “EF‐1340921”).

## Conflict of Interest

None declared.
